# IL-34 Suppresses *Candida albicans* Induced TNF*α* Production in M1 Macrophages by Downregulating Expression of Dectin-1 and TLR2

**DOI:** 10.1155/2015/328146

**Published:** 2015-06-03

**Authors:** Rong Xu, Hong-Fan Sun, David W. Williams, Adam V. Jones, Ali Al-Hussaini, Bing Song, Xiao-Qing Wei

**Affiliations:** ^1^Tissue Engineering and Reparative Dentistry, School of Dentistry, Cardiff University, Heath Park, Cardiff CF14 4XY, UK; ^2^Tianjin Key Laboratory of Biomaterial Research, Institute of Biomedical Engineering, Chinese Academy of Medical Science, Tianjin 300192, China

## Abstract

*Candida albicans* is a fungus that is an opportunistic pathogen of humans. Normally,* C. albicans* exists as a harmless commensal and does not trigger inflammatory responses by resident macrophages in skin mucosa, which may be caused by a tolerance of skin macrophage to* C. albicans*. IL-34 is a recently discovered cytokine, constitutively expressed by keratinocytes in the skin. IL-34 binds to the receptor of M-CSF, thereby stimulating tissue macrophage maturation and differentiation. Resident macrophages exhibit phenotypic plasticity and may transform into inflammatory M1 macrophages for immunity or anti-inflammatory M2 macrophages for tissue repair. M1 macrophages produce higher levels of inflammatory cytokines such as TNF*α* in response to* C. albicans* stimulation. In this study, it was demonstrated that IL-34 attenuated TNF*α* production by M1 macrophages challenged with heat killed Candida (HKC). The molecular mechanism of IL-34 mediated suppression of HKC induced TNF*α* production by M1 macrophages was by the inhibition of M1 macrophage expression of key* C. albicans* pattern recognition receptors (PPRs), namely, Toll-like receptor (TLR) 2 and Dectin-1. The results of this study indicated that constitutive IL-34 expressed by skin keratinocytes might suppress resident macrophage responses to *C. albicans* colonisation by maintaining low levels TLR2 and Dectin-1 expression by macrophages.

## 1. Introduction

*Candida albicans* is an opportunistic fungal pathogen that can cause life-threatening infections in severely immunocompromised individuals. However,* C. albicans* is a frequent coloniser of mucosal surfaces in the majority of healthy humans without causing disease. This commensal behaviour has been associated with host immune tolerance [1, 2]. Key immune surveillance cells in the mucosa that recognise and respond to* C. albicans* are resident macrophages, such as the Langerhans cells of mucosal and dermal skin [3, 4]. Resident macrophages exhibit phenotypic plasticity [5]. M1 macrophages may control* Candida* infection through phagocytosis and also induction of inflammation in the skin by producing inflammatory cytokines, such as tumour necrosis factor alpha (TNF*α*) [6–8]. M2 macrophages may also kill* Candida* by phagocytosis, but, importantly, can also produce anti-inflammatory cytokines and growth factors leading to immune tolerance to* Candida* [1, 9].

*β*-glucan is a major cell wall component of* C. albicans* and binds to Toll-like receptor (TLR) 2 and Dectin-1 receptors on M1 macrophages to trigger downstream cell signaling for TNF*α* production [10–12]. We have previously reported the Granulocyte-Macrophage Colony-Stimulating Factor (GM-CSF) mediated stimulation of Dectin-1 expression by human peripheral blood monocytes [13]. This in turn promoted M1 macrophage maturation,* Candida* phagocytosis, and TNF*α* production [14, 15]. M1 macrophages are normally found at sites of inflammation of the skin and are associated with GM-CSF expression by infiltrating and reactive CD4^+^ T cells, resulting in higher levels of TNF*α* [1, 16, 17]. We have also shown that denture stomatitis involving* C. albicans* is associated with higher levels of TNF*α* in fluids from the oral palatal mucosa [18].

IL-34 is a recently identified cytokine that binds to the macrophage colonisation factor (M-CSF) receptor (c-fms or CD115) to stimulate macrophage expansion and maturation. IL-34 expression occurs predominantly in the skin and brain where it is produced by keratinocytes and neuronal cells, respectively [19]. Importantly, mice devoid of IL-34 exhibit a loss of Langerhans cells in the skin and have a reduced number of microglia in the brain [20, 21]. The Langerhans cells and resident macrophages in mucosal and dermal skin of healthy individuals do not normally respond to* C. albicans* or other commensal microorganisms and in these cases there is no inflammation [22]. Such tolerance could arise through the presence of locally produced cytokines, such as IL-34.

In the present study, we have examined the potential role of IL-34 in inducing immune tolerance by resident macrophages towards heat killed* Candida* (HKC). This was undertaken by measuring induced TNF*α* production and elucidation of molecular mechanisms. The results have demonstrated, for the first time, that IL-34 acts as an immune suppressing cytokine which may maintain mucosal and dermal skin tolerance to* C. albicans* by downregulating TLR2 and Dectin-1 expression by M1 macrophages, resulting in lower TNF*α* production.

## 2. Material and Methods

### 2.1. Macrophage Culture and Stimulation

Bone marrow derived macrophages (BMMΦs) were isolated from 6- to 8-week-old C57BL/6 mice as previously described [1] and cultured with 10 ng/mL GM-CSF (R&D) for 7 days to drive M1 macrophage maturation. The mouse macrophage cell line, RAW264.7, was also treated with GM-CSF (10 ng/mL) for 2 days to induce M1-like macrophages. All cells were maintained at 37°C in a 5% (v/v) CO_2_ enriched atmosphere in RPMI 1640 medium, supplemented with 10% (v/v) fetal bovine serum (FBS) containing penicillin and streptomycin.

To investigate TNF*α* production by mouse macrophages, mature M1 BMMΦs or RAW264.7 cells were first seeded at levels of 2 × 10^5^ cells in the wells of a 24-well tissue culture plate. To these cells, IL-34 (R&D Systems) was added at 0, 10, or 100 ng/mL, and cells were then incubated with the added presence of 10 ng/mL GM-CSF for 5 (mature M1 BMMΦs) or 2 (RAW264.7 cells) days. After incubation, 1 × 10^5^ HKC, which had been prepared as previously described [1, 13], was added. Wells devoid of HKC served as negative controls. Cell culture supernatants were collected 24 h after stimulation and TNF*α* was detected using an enzyme-linked immunosorbent assay (ELISA).

### 2.2. Detection of Toll-Like Receptor 2 (TLR2) and Dectin-1 in Mouse Macrophages

To detect TLR2 and Dectin-1 expression by M1 BMMΦs and RAW264.7, cells were treated with IL-34 at 10 or 100 ng/mL for M1 BMMΦs and RAW264.7, respectively, in the presence of GM-CSF. Cells not exposed to IL-34 served as controls. M1 BMMΦs were harvested at 0, 24, 48, 72, and 96 h of culture, whilst RAW264.7 cells were harvested at 6, 12, and 24 h. Total RNA was extracted from all harvested cells and TLR2 and Dectin-1 expression was then measured by real-time reverse transcription-polymerase chain reaction (RT-PCR) and in the case of RAW264.7 cells also by fluorescence-activated cell sorting (FACS).

### 2.3. TNF*α* ELISA

TNF*α* in cell culture medium was measured by ELISA according to the manufacturer's (eBioscience) instructions. Briefly, the wells of high protein bound 96-well plates (Nunc) were coated overnight at 4°C with 50 *μ*L of anti-mouse TNF*α* specific antibody (0.5 *μ*g/mL) in phosphate buffered saline (PBS). After blocking nonspecific binding using an assay diluent (supplied in the ELISA kit) for 2 h at room temperature (RT), cell-free supernatants (50 *μ*L) and mouse recombinant TNF*α* standards (from 4 ng/mL in two-fold dilutions) were added to the plate in triplicate prior to overnight incubation at 4°C to capture TNF*α*. After thoroughly washing the plate with PBS containing 0.05% (v/v) Tween-20, specific bound TNF*α* was detected using a biotin-conjugated anti-mouse TNF*α* antibody (0.5 *μ*g/mL) (1 h at RT), followed by incubation with 50 *μ*L of StreptAvidin-HRP for 30 min at RT. A 50-*μ*L volume of 3,3′,5,5′-tetramethylbenzidine (TMB) peroxidase substrate was added to each well and incubated at room temperature for 10–20 min for color development. The enzyme reaction was terminated by adding 50 *μ*L of a stop solution (supplied with the kit). The optical density of each well was then measured with a spectrometer at 450_nm_ wavelength and the TNF*α* concentration in the supernatants determined using a standard curve.

### 2.4. Real-Time RT-PCR

Total RNA was purified using the RNeasy Mini Kit with QIAshredder spin columns (QIAGEN) and transcribed into cDNA following the manufacturer's recommended protocol using Avian Myeloblastosis Virus Reverse Transcriptase (AMV RT) (Promega) and 50 ng of random primers in a total volume of 25 *μ*L for 1 h at 37°C. The SYBR green qPCR kit (Sigma) was used to quantify mRNA levels of TLR2, TLR4, Dectin-1, and Mannose Receptor (MR) using the ABI PRISM 7000 Sequence Detection System (Applied Biosystems).  Table 1 lists the primer pairs that were used to measure mouse receptor mRNA levels compared with the mouse housekeeping *β*-actin gene (Table 1).

All samples were run in triplicate. The cycle threshold (CT) value of each sample was determined by calculation of 2^−ΔΔ^CT, and the data was presented as an expression-fold relative to control.

### 2.5. FACS Analysis

RAW264.7 cells, with or without IL-34 treatment, were harvested at specific time points and fixed in 300 *μ*L of FACS buffer containing 2% (v/v) paraformaldehyde for 30 min at 4°C. After blocking nonspecific binding with 5% (v/v) rabbit serum for 20 min at 4°C, cells were incubated either with FITC-conjugated anti-TLR2 antibody (eBioscience), Phycoerythrin- (PE-) conjugated anti-Dectin-1 antibody (eBioscience), or isotype control antibodies diluted in FACS buffer for 30 min at 4°C. Samples were analysed by collecting 10,000 events using the FACSCanto II flow cytometer (BD Biosciences). The change in percentage of both TLR2 and Dectin-1 positive cells after treatment with IL-34 was determined and compared to cells without IL-34 treatment.

### 2.6. Statistical Analysis

Results were calculated as means ± standard deviations (SD). Statistical significance was determined using two-tailed Student's *t*-test and Tukey-Kramer or Bonferroni multiple comparisons post-tests to analyse differences between groups; ^*∗*^ *P* < 0.05 and ^*∗∗*^ *P* < 0.01 were considered significant.

## 3. Results

### 3.1. IL-34 Suppressed HKC Induced TNF*α* Production in M1 Macrophages in a Dose Dependent Manner

**Figure 1:**
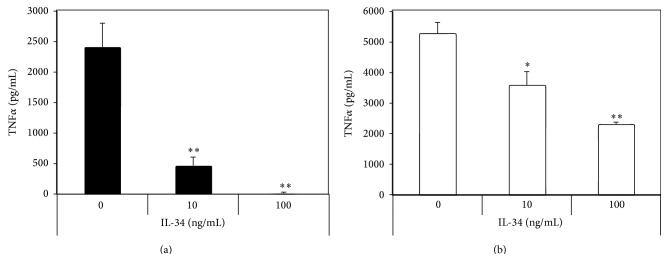
IL-34 suppressed HKC induced TNF*α* production in M1 macrophages. (a) IL-34 dose dependent suppression of HKC induced TNF*α* production in mouse bone marrow derived M1 macrophages. The differences of induced TNF*α* production between 10 ng/mL and 100 ng/mL of IL-34 were also significant (*P* < 0.05). (b) IL-34 suppression of HKC induced TNF*α* production in M1 macrophage like cell line, derived from RAW264.7 cells. ^*∗*^ *P* < 0.05 and ^*∗∗*^ *P* < 0.01.

HKC stimulated TNF*α* production by M1 macrophages, which is one of the phenotypic features of M1 macrophages [1]. To assess the effect of IL-34 on the inflammatory M1 macrophage, M1 macrophages were pretreated with different concentrations of IL-34 for 5 days in the presence of GM-CSF, prior to the cells being stimulated with HKC. Decreased TNF*α* production in culture supernatant was determined by ELISA in a HKC dose dependent manner (Figure 1(a)). These results were confirmed using the cultured mouse macrophage cell line (RAW264.7). However, treatment of RAW264.7 cells with GM-CSF for two days, followed by 2 days treatment with IL-34, demonstrated suppression towards HKC induced TNF*α* production in an IL-34 dose dependent manner (Figure 1(b)). This result showed that IL-34 was acting as an immune suppressing cytokine that was able to inhibit TNF*α* production by inflammatory M1 macrophages in response to* Candida* stimulation.

### 3.2. IL-34 Inhibited TLR2 and Dectin-1 mRNA Expression by M1 Macrophages

**Figure 2:**
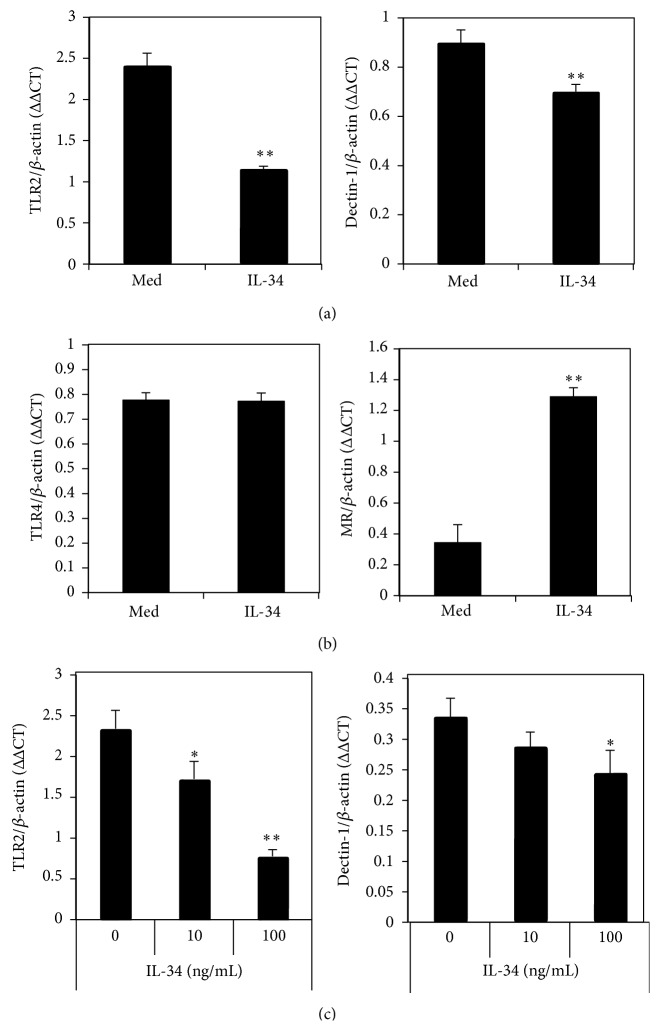
IL-34 suppressed TLR2 and Dectin-1 mRNA expression in M1 macrophages. M1 macrophages derived from mouse bone marrow cells were cultured with or without mouse recombinant IL-34 (100 ng/mL). (a) The mRNA expression of TLR2 and Dectin-1 was decreased. (b) TLR4 expression was not altered by IL-34. However, Mannose Receptor (MR) was significantly upregulated by IL-34. (c) IL-34 suppressing TLR2 and Dectin-1 was confirmed in RAW264.7 cell lines in a dose dependent manner. The results are the representative of 3 experiments. ^*∗*^ *P* < 0.05 and  ^*∗∗*^ *P* < 0.01.

The recognition of* Candida* cell wall components by macrophages involves Toll-like receptors (TLRs, mainly TLR2 and TLR4) and C-type lectin receptors (Dectin-1 and Mannose Receptor). To investigate the molecular mechanism of IL-34 suppression of* Candida* induced TNF*α* production by M1 macrophages, the mRNAs of TLR2, TLR4, Dectin-1, and Mannose Receptor were measured by RT-PCR. IL-34 treated cells expressed significantly lower mRNA for TLR2 and Dectin-1 (*P* < 0.01) (Figure 2(a)). There was, however, no significant decrease in the expression of mRNA for TLR4 (Figure 2(b)). Interestingly, Mannose Receptor was significantly upregulated by IL-34 in M1 macrophages (Figure 2(b)). IL-34 suppression of TLR2 and Dectin-1 mRNA was confirmed using RAW264.7 cells (Figure 2(c)). These results showed that IL-34 suppression of HKC stimulated TNF*α* production was via the downregulation of TLR2 and Dectin-1 expression by M1 macrophages and not by the downregulation of TLR4 or Mannose Receptor.

### 3.3. Detection of Reduced TLR2 and Dectin-1 Cell Surface Expression in M1 Macrophages

**Figure 3:**
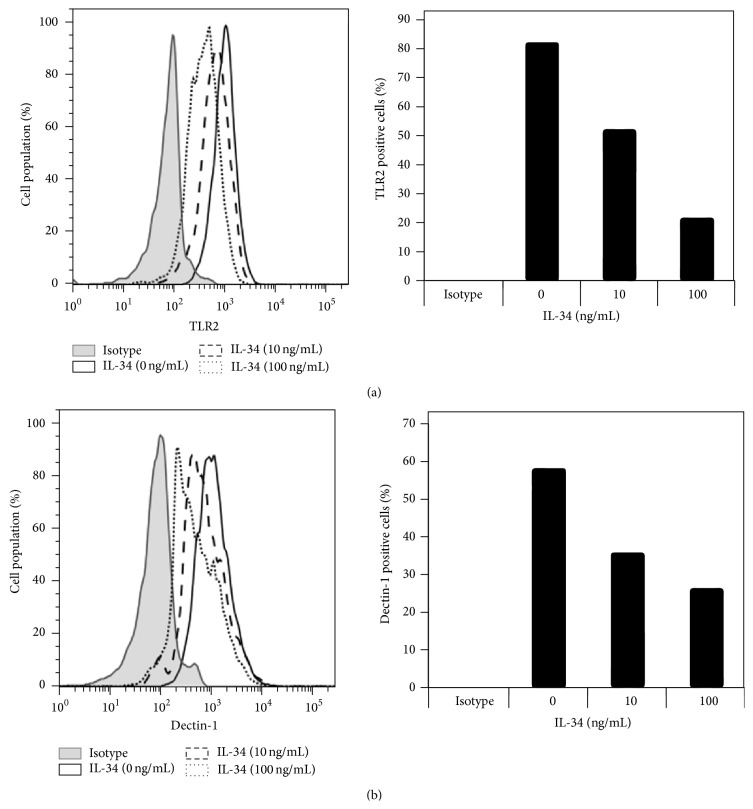
IL-34 suppressed cell surface expression of TLR2 and Dectin-1 in bone marrow derived M1 macrophages. Cell surface expression of TLR2 and Dectin-1 decreased with increasing IL-34 levels in cultures. The bar figures showed percentage changes with positive cell staining for TLR2 and Dectin-1, respectively. Isotype staining was used as negative controls. The results are representative of 3 independent experiments.

TLR2 and Dectin-1 cell surface expression can be detected by FACS analysis using anti-TLR2 and anti-Dectin-1 receptor specific antibodies. To confirm downregulation of cell surface TLR2 and Dectin-1 following IL-34 stimulation, M1 macrophages treated with different concentrations of IL-34 were stained with FITC-conjugated anti-TLR2 and PE-conjugated anti-Dectin-1. A dose dependent decrease in TLR2 (Figure 3(a)) and Dectin-1 (Figure 3(b)) positive cell populations of M1 macrophages was demonstrated by FACS analysis. These results further demonstrated that IL-34 treatment of M1 macrophage cell cultures reduced cell surface expression of TLR2 and Dectin-1, resulting in inhibition of* Candida* induced TNF*α* production by M1 macrophages.

## 4. Discussion

Mucosal and dermal skin provides a physical and biological barrier against insult from both the environment and pathogens. These surfaces also exhibit tolerance to commensal microorganisms such as* C. albicans* that colonise them. This immune tolerance arises as a result of a complex interaction of cell components including tissue resident macrophages such as Langerhans cells in the epidermal layers of skin [22, 23]. Tissue resident macrophages exhibit phenotypic plasticity. GM-CSF induces an inflammatory M1 macrophage phenotype* in vitro* and* in vivo*, and M1 macrophages produce TNF*α* in response to* C. albicans* stimulation [6–8].

In this study, we demonstrated that IL-34, a cytokine constitutively produced in the skin, was able to suppress inflammatory M1 macrophages for HKC induced TNF*α* production. This suppressing activity was demonstrated for M1 macrophages cultured in the presence of GM-CSF, which indicated that IL-34 was an antagonist of GM-CSF. Recent studies have shown that Langerhans cells exhibit immune tolerance and do not normally induce an immune response even after activation and migration to a draining lymph node [22]. This immune tolerance may be the result of an IL-34 rich environment in the skin. The evidence for the immune tolerant phenotype of Langerhans cells and IL-34 inducing non-TNF*α* producing macrophages suggests that IL-34 is an immune suppressing cytokine of the skin. However, studies of autoimmune rheumatoid arthritis have indicated that IL-34 may be a proinflammatory cytokine with expression associated with joint inflammation [24–28]. IL-34 shares the receptor for M-CSF, which is a key factor in inducing osteoclast formation. Indeed, it has been demonstrated that IL-34 has involvement in cartilage and bone erosion by promoting osteoclastogenesis in rheumatoid arthritis [25, 26]. Although it has been shown that increasing IL-34 levels is associated with synovitis severity of rheumatoid arthritis patients [24], IL-34 expression could suppress joint inflammation as a negative feedback instead of promoting inflammation. Studies have also shown that suppressing M1 macrophages reduces collagen induced arthritis in mice [29]. It has been demonstrated that CD14^+^ macrophages in synovial tissue are a major source of TNF*α* [30]. The role of IL-34 in joint inflammation may need to be analysed with greater focus given to the impact of this cytokine on M1 macrophages in synovial tissues of patients.

TNF*α* is a key proinflammatory cytokine in both skin and joint inflammation. We have recently demonstrated that higher TNF*α* production and inflammation in oral mucosa was associated with* C. albicans* infection in denture stomatitis [18]. Following sufficient control of* C. albicans* colonisation in such patients, a reduction of M1 macrophage activity using IL-34 might therefore be beneficial.

*Candida albicans* stimulation of macrophages leading to TNF*α* production requires expression of the pattern recognition receptors, TLR2 and Dectin-1 [10–12, 31]. In the current study, we have shown for the first time that IL-34 can suppress both TLR2 and Dectin-1 mRNA transcription, but not transcription of TLR4 and Mannose Receptor. Both cell surface TLR2 and Dectin-1 expression was also suppressed by IL-34 in a dose dependent manner. These findings indicate that TLR2 and Dectin-1 (but not TLR4 and Mannose Receptor) are the cell surface receptors responsible for TNF*α* production by M1 macrophages. IL-34 expression is restricted to the epidermal layers of skin and certain areas of the brain [19]. Regeneration of Langerhans cells in mucosal and dermal skin is entirely dependent on IL-34 production in keratinocytes. Loss of Langerhans cells occurs in IL-34 deficient mice indicating that the function of IL-34 cannot be undertaken by M-CSF, despite IL-34 and M-CSF sharing a common receptor (c-fms or CD115) in macrophage precursor cells. The results of the present study provide evidence that constitutive expression of IL-34 in the skin may maintain an environment of immune tolerance for tissue resident macrophages. In this study, IL-34 stimulated a significantly higher Mannose Receptor (CD206) expression, which is a cell marker for M2 macrophages and responsible for phagocytosis. This result further demonstrated that IL-34 was able to promote M1 to M2 macrophage transformation, which could be of benefit in generating immune tolerance and wound healing in the skin.

In conclusion, this study demonstrated that IL-34 suppresses* C. albicans* induced TNF*α* production by GM-CSF induced M1 macrophages. The mechanism of inhibition was through the downregulation of TLR2 and Dectin-1 expression by M1 macrophages, but not TLR4 and Mannose Receptor. Constitutive IL-34 expression in keratinocytes in both mucosal skin and dermal skin may play a key role in maintaining immune tolerance toward commensal colonising microorganisms.

## Figures and Tables

**Table 1:** Primers used for real-time RT-PCR analysis of mouse receptor mRNA.

mDectin-1-P1	TGGTAGTAGTGGTTGCTGCAGTGCTGGG
mDectin-1-P2	GTAGTTTGGGATGCCTTGGAGGGAGCCA
mMR-P1	GGTACACTAACTGGGGTGCTGACGAGCC
mMR-P2	ACTCTGGACACTTGCCAGGCAGTTGAGG
mTLR4-P1	GGCACTGTTCTTCTCCTGCCTGACACCA
mTLR4-P2	AGGGACTTTGCTGAGTTTCTGATCCATGC
mTLR2-P1	GGAGCATCCGAATTGCATCACCGGTCAGA
mTLR2-P2	GGCCATCACACACCCCAGAAGCATCACA
M*β*-actin-P1	CTTCTTTGCAGCTCCTTCGTTGCCGGT
M*β*-actin-P2	CCTTCTGACCCATTCCCACCATCACACC

## References

[1] Zheng X.-F., Hong Y.-X., Feng G.-J. (2013). Lipopolysaccharide-induced M2 to M1 macrophage transformation for IL-12p70 production is blocked by *Candida albicans* mediated up-regulation of EBI3 expression. *PLoS ONE*.

[2] Jouault T., Sarazin A., Martinez-esparza M., Fradin C., Sendid B., Poulain D. (2009). Host responses to a versatile commensal: PAMPs and PRRs interplay leading to tolerance or infection by *Candida albicans*. *Cellular Microbiology*.

[3] Romani L. (2011). Immunity to fungal infections. *Nature Reviews Immunology*.

[4] Netea M. G., Brown G. D., Kullberg B. J., Gow N. A. R. (2008). An integrated model of the recognition of *Candida albicans* by the innate immune system. *Nature Reviews Microbiology*.

[5] Giorgio S. (2013). Macrophages: plastic solutions to environmental heterogeneity. *Inflammation Research*.

[6] Xu G., Liu G., Xiong S., Liu H., Chen X., Zheng B. (2015). The histone methyltransferase Smyd2 is a negative regulator of macrophage activation by suppressing interleukin 6 (IL-6) and tumor necrosis factor *α* (TNF-*α*) production. *The Journal of Biological Chemistry*.

[7] Cabrera-Fuentes H. A., Lopez M. L., McCurdy S. (2015). Regulation of monocyte/macrophage polarisation by extracellular RNA. *Thrombosis and Haemostasis*.

[8] Hamilton T. A., Zhao C., Pavicic P. G., Datta S. (2014). Myeloid colony-stimulating factors as regulators of macrophage polarization. *Frontiers in Immunology*.

[9] Akilbekova D., Philiph R., Graham A., Bratlie K. M. (2014). Macrophage reprogramming: influence of latex beads with various functional groups on macrophage phenotype and phagocytic uptake in vitro. *Journal of Biomedical Materials Research Part A*.

[10] Takahara K., Tokieda S., Nagaoka K., Inaba K. (2012). Efficient capture of *Candida albicans* and zymosan by SIGNR1 augments TLR2-dependent TNF-*α* production. *International Immunology*.

[11] Netea M. G., Gow N. A. R., Munro C. A. (2006). Immune sensing of *Candida albicans* requires cooperative recognition of mannans and glucans by lectin and Toll-like receptors. *The Journal of Clinical Investigation*.

[12] Underhill D. M., Ozinsky A., Hajjar A. M. (1999). The Toll-like receptor 2 is recruited to macrophage phagosomes and discriminates between pathogens. *Nature*.

[13] Rogers H., Williams D. W., Feng G.-J., Lewis M. A. O., Wei X.-Q. (2013). Role of bacterial lipopolysaccharide in enhancing host immune response to *Candida albicans*. *Clinical and Developmental Immunology*.

[14] Holmkvist P., Roepstorff K., Uronen-Hansson H. (2014). A major population of mucosal memory CD4^+^ T cells, coexpressing IL-18R*α* and DR3, display innate lymphocyte functionality. *Mucosal Immunology*.

[15] Sindrilaru A., Scharffetter-Kochanek K. (2013). Disclosure of the culprits: macrophages-versatile regulators of wound healing. *Advances in Wound Care*.

[16] Leon F., Contractor N., Fuss I. (2006). Antibodies to complement receptor 3 treat established inflammation in murine models of colitis and a novel model of psoriasiform dermatitis. *The Journal of Immunology*.

[17] Nonaka K., Saio M., Suwa T. (2008). Skewing the Th cell phenotype toward Th1 alters the maturation of tumor-infiltrating mononuclear phagocytes. *Journal of Leukocyte Biology*.

[18] Roger H., Wei X.-Q., Lewis M. A. O. (2013). Immune response and candidal colonisation in denture associated stomatitis. *Journal of Clinical & Cellular Immunology*.

[19] Wang Y., Colonna M. (2014). Interkeukin-34, a cytokine crucial for the differentiation and maintenance of tissue resident macrophages and Langerhans cells. *European Journal of Immunology*.

[20] Wang Y., Szretter K. J., Vermi W. (2012). IL-34 is a tissue-restricted ligand of CSF1R required for the development of Langerhans cells and microglia. *Nature Immunology*.

[21] Greter M., Lelios I., Pelczar P. (2012). Stroma-derived interleukin-34 controls the development and maintenance of langerhans cells and the maintenance of microglia. *Immunity*.

[22] Shklovskaya E., O'Sullivan B. J., Ng L. G. (2011). Langerhans cells are precommitted to immune tolerance induction. *Proceedings of the National Academy of Sciences of the United States of America*.

[23] Tay S. S., Roediger B., Tong P. L., Tikoo S., Weninger W. (2014). The skin-resident immune network. *Current Dermatology Reports*.

[24] Chemel M., Le Goff B., Brion R. (2012). Interleukin 34 expression is associated with synovitis severity in rheumatoid arthritis patients. *Annals of the Rheumatic Diseases*.

[25] Hwang S.-J., Choi B., Kang S.-S. (2012). Interleukin-34 produced by human fibroblast-like synovial cells in rheumatoid arthritis supports osteoclastogenesis. *Arthritis Research and Therapy*.

[26] Masteller E. L., Wong B. R. (2014). Targeting IL-34 in chronic inflammation. *Drug Discovery Today*.

[27] Moon S. J., Hong Y. S., Ju J. H., Kwok S. K., Park S. H., Min J. K. (2013). Increased levels of interleukin 34 in serum and synovial fluid are associated with rheumatoid factor and anticyclic citrullinated peptide antibody titers in patients with rheumatoid arthritis. *Journal of Rheumatology*.

[28] Tian Y., Shen H., Xia L., Lu J. (2013). Elevated serum and synovial fluid levels of interleukin-34 in rheumatoid arthritis: possible association with disease progression via interleukin-17 production. *Journal of Interferon and Cytokine Research*.

[29] Li J., Hsu H. C., Ding Y. (2014). Inhibition of fucosylation reshapes inflammatory macrophages and suppresses type II collagen-induced arthritis. *Arthritis & Rheumatology*.

[30] Gracie J. A., Forsey R. J., Chan W. L. (1999). A proinflammatory role for IL-18 in rheumatoid arthritis. *Journal of Clinical Investigation*.

[31] Gil M. L., Gozalbo D. (2006). TLR2, but not TLR4, triggers cytokine production by murine cells in response to Candida albicans yeasts and hyphae. *Microbes and Infection*.

